# Ameliorative effects of omega-3 against profenofos-induced neurotoxicity in rats via PI3K/AKT pathway

**DOI:** 10.1038/s41598-026-42757-9

**Published:** 2026-04-06

**Authors:** Ahmed Medhat Hegazy, Rania M. Elbatawy, Lina Abdelhady Mohammed, Naglaa Rafaat, Dania Abdelhady

**Affiliations:** 1 Department of Forensic Medicine and Toxicology, Faculty of Veterinary Medicine, Benha University, Moshtohor, Toukh, 13736 Qalyubia Egypt; 2 Department of Pathology, Faculty of Veterinary Medicine, Benha University, Moshtohor, Toukh, 13736 Qalyubia Egypt; 3 Department of Medical Biochemistry and Molecular Biology, Faculty of Medicine, Benha University, Benha, 13518 Egypt; 4 Department of Biomedical Sciences (Biochemistry), Dubai Medical University, Dubai, United Arab Emirates; 5 Department of Medical Biochemistry and Molecular Biology, Faculty of Medicine, Ain Shams University, Cairo, Egypt; 6 Department of Physiology, Faculty of Medicine, Benha University, Benha, 13518 Egypt

**Keywords:** Apoptosis, neuroprotection, NFқB/STAT-3 inflammasomes, omega-3, oxidative stress, profenofos, Biochemistry, Drug discovery, Neuroscience, Physiology

## Abstract

Profenofos (PFF) is a class of organophosphate insecticides and acaricides. The poison is mainly neurotoxic that causes pest death by affecting acetylcholinesterase activity. The health of the brain is significantly influenced by omega-3 (long chain polyunsaturated fatty acids). It is mostly found in fish rich in fat. Although omega-3 has been shown in numerous studies to have anti-inflammatory and antioxidant properties in the brain, it is unknown how it affects PI3K/AKT-mediated apoptosis in brain toxicity. In Wistar rats subjected to PFF-induced neurotoxicity, the current study sought to evaluate the ameliorative effects of omega-3 on brain function (cerebral cortex), oxidative stress, and PI3K/AKT-mediated apoptosis. Twenty-eight male Wistar rats were divided into four equal groups. The following were the groups of rats: Group 1 (G1) remained a healthy control group. For four weeks, G2 received daily omega-3 at a dose of 100 mg/kg b.wt. G3 received PFF at a dose of 35 mg/kg b.wt. twice a week. G4 received an omega-3 at the same dose and duration one hour prior to the PFF injection. The findings demonstrated that omega-3 reduces the brain dysfunctions brought on by PFF by reestablishing the levels of MDA, GSH, SOD, triglycerides, cholesterol, and cholinesterase activity. Furthermore, the pro-inflammatory NFκB/STAT-3 mRNA gene expression was repressed and the anti-inflammatory PI3K/AKT mRNA gene expression was stimulated. In addition, Caspase-3 (Cas-3) and Tumor necrosis factor-alpha (TNF-α) immunohistochemistry area% were markedly lower with only a limited number of neurons displaying mild to moderate immunostaining, indicating a notable reduction in apoptotic glial cells and inflammation. By lessening the degenerative alterations, omega-3 also lessens the impact of inflammation on the cerebral cortex. In conclusion, omega-3 has the potential to ameliorate PFF sub-acute toxicity induced brain dysfunction by targeting PI3K/AKT pathway.

## Introduction

The use of pesticides poses serious risks to human health because they have permanently infiltrated our atmosphere and tainted food, water, and soil^1^. Organophosphorus pesticides (OPs) have been widely employed to manage household and agricultural pests^2^. There have been reports of OPs exposure among agricultural workers and their families^3^. OPs are attacked by soil bacteria, which can rapidly break down these insecticides when they hydrolyze in the presence of sunlight and air. Nonetheless, it is observed to remain in food and water in trace concentrations^4^. Profenofos (PFF) [O-(4-bromo-2-chlorphenyl) O-ethyl S-propyl phosphonothioate] is an OPs of acaricides and insecticides^5^. It causes paralysis and pest death by affecting the acetylcholinesterase activity^6^. PFF exposure can cause endocrine disruption, genotoxicity, developmental and reproductive toxicity, and multi-organ dysfunction^7^.

On the molecular level; Cell growth, metabolism, and survival depends on the anti-inflammatory PI3K/AKT genes. Inhibited AKT signaling enhancing mitochondrial dysfunction, and reduce cellular repair^8^. On the other side, the pro-inflammatory NFκB gene regulates inflammatory responses, apoptosis, and stress signaling^9^. In addition, the STAT-3 gene regulates cell growth, differentiation, inflammation, and survival^10^. PFF increases the generation of reactive oxygene species (ROS), which attack membrane lipids leading to lipid peroxidation. PFF can aberrantly activate NFκB, possibly through ROS. This leads to transcription of pro-inflammatory and pro-apoptotic genes that increased cellular damage in brain^1^.

The health of the brain is significantly influenced by omega-3 long chain polyunsaturated fatty acids^11^. Omega-3 fatty acids have an impact on transmembrane ion channels, cell membrane function, and physiological process regulation^12^. Eicosapentaenoic acid (EPA) is often found in relatively small concentrations in the CNS, whereas docosahexaenoic acid (DHA) is the most prevalent omega-3 fatty acid inside phospholipids of brain cell membranes^13^. Consuming fatty fish continues to be the main source of long-chain polyunsaturated fatty acids (DHA and EPA)^14^. By reducing oxidative stress, proinflammatory mediators, and lipid peroxidation, DHA incorporation into cell membranes shields brain cells^15^. The effect of omega-3 on the PI3K/AKT pathway in brain cells still unclear. Therefore, the goal of this study was to evaluate on how omega-3 fatty acids protects Wistar rats against subacute cerebral cortex intoxication induced by PFF by targeting the PI3K, AKT, NFκB, STAT-3 genes.

## Materials and methods

### Chemicals

Profenofos (PFF) (Deliron El-Nasr^®^) 72% (emulsifiable concentrate) was obtained from El-Nasr Company for Chemicals, (Cairo, Egypt). To get the required concentration, PFF was dissolved in D.W. The supplier of omega-3 capsules was SEDICO Pharmaceutical, (Cairo, Egypt). Each soft gelatin omega-3 capsule contains fish Oil 1000 mg [Eicosapentaenoic acid (EPA) 13% and Docosahexaenoic acid (DHA) 9%]. To get the required concentration, it was dissolved in phosphate buffer saline.

### Experimental animals

Male adult Wistar rats that were eight weeks old and weighed between 150 and 180 g were acquired from the National Research Center’s animal house (Dokky, Giza, Egypt). Rats were kept in neat cages. Rats were kept in a typical environment with free access to food and water, a 12-hour light/dark cycle, and a room temperature of 23 °C. All groups had baseline body weight measurement taken prior to the therapy. To adjust the chemical dosage, weekly animal weight were recorded.

### Experimental design

From 28 rats, four equal groups of seven adult male Wistar rats each were formed. As a healthy control group, the control group (G1) was kept. The second group (G2) was intragastric (I/G) administered omega-3 100 mg/kg b.wt. in phosphate buffer saline daily for 4 weeks (0.5mL/rat)^16^. The third group (G3) was I/G administered PFF 35 mg/kg b.wt. in D.W. twice/week (0.5mL/rat)^7^. Omega-3 was given to the fourth group (G4) at the same dose and duration one hour prior to PFF. After the study was finished, the experimental rats were put to euthanize and anesthesia with isoflurane inhalation before being beheaded. Blood samples were then taken to estimate the lipid profile and acetylcholinesterase activity, and brain tissue samples (cerebral cortex) were taken to evaluate oxidative markers, gene expression, and histopathological alterations.

### Brain homogenate preparation

Brain tissue (cerebral cortex) homogenates were made using the procedures described by Hegazy et al.^17^. For the collected supernatant, measurements were made of the oxidative markers [lipid peroxidation byproduct (MDA) level, reduced glutathione (GSH) level, and superoxide dismutase (SOD) activity], and total protein levels.

### Assay methods

#### Lipid profile

The method described by Cox and García-Palmieri^18^, was used to measure the serum triglycerides (TG), total cholesterol (TC), and high-density lipoprotein cholesterol (HDL-C). The method of Martin et al.^19^ was used to determine the serum low-density lipoprotein cholesterol (LDL-C).

#### Cholinesterase activity

Cholinesterase activity was determined using the method of Ellman et al.^20^.

#### Oxidative indicators in homogenates of brain tissue

The levels of superoxide dismutase (SOD)^21^, reduced glutathione (GSH)^22^, malondialdehyde (MDA)^23^, and the total protein content^24^ were determined. A spectrophotometer (UV/VIS, Model JASCO 7800, Japan) was used for measurement.

### Expression of mRNA genes in the brain

Real-time polymerase chain reaction (PCR) was used to assess the mRNA expression for the phosphoinositide 3-kinase (PI3K), protein kinase B (AKT), nuclear factor kappa B (NFκB), and signal transducer and activator of transcription (STAT-3) genes. The PI3K, AKT, NFκB, STAT-3, and GAPDH primer sets were illustrated in Table 1. An Applied Biosystems 7300 real-time PCR system (Foster City, California, USA) was used for thermal cycling and fluorescence detection. The sample was run in triplicate different wells on the same plate. Amplification conditions included 15 min at 95 °C, followed by 40 cycles at 95 °C for 15 s, at 60–63 °C for 15 s and 72 °C for 30 s. Melting curve analysis was conducted following each real-time PCR and analyzed using the 2^−ΔΔCt^ method. To identify variations in gene expression, cycle threshold (Ct) values obtained from real-time PCR were applied to a reference (housekeeping) gene (GAPDH)^25^.

**Table 1:** The primer sets of the assessed genes.

Genes	Forward primer (Sense)	Reverse primer (antisense)	Accession no
PI3K	5′-TCAACCGAAACTCCTCCAGC-3′	5′-GGAAAGGGTTGTTGTTGCCC-3′	NM_053481.2
AKT	5′-GAAGGAGAAGGCCACAGGTC-3′	5′-TTCTGCAGGACACGGTTCTC-3′	NM_033230.3
NFқB	5′-GCACCCCACCATCAAGATCA-3′	5′- CACACTGGATCCCCAGGTTC-3′	NM_199267.2
STAT-3	5′-CTGCCCCTTACCTGAAGACC-3′	5′-TCCATGTCAAACGTGAGCGA-3′	NM_001430048.1
GAPDH	5′-ACGGGAAACCCATCACCATC-3′	5′-TCACAAACATGGGGGCATCA-3′	NM_017008.4

### Histopathological examination

In all groups, the cerebral cortex was immediately excised, rinsed gently in cold physiological saline, and fixed for a day in 10% neutral buffered formalin. The fixed tissues underwent standard histological processing, including dehydration in ascending grades of ethanol, clearing in xylene, and embedding in paraffin wax. Paraffin blocks were sectioned at 4–5 μm thickness using a rotary microtome, and the sections were mounted on glass slides and stained with hematoxylin and eosin (H&E) for microscopic evaluation. All stained sections were examined under a light microscope (Eclipse E800, Nikon) and photographed using an Olympus camera^26^.

### Immunohistochemical study

For immunohistochemical evaluation of apoptosis and inflammation, Caspase-3 (Cas-3) and Tumor necrosis factor-alpha (TNF-α) expression were assessed respectively, in the cerebral cortex from all experimental groups. Paraffin-embedded tissue blocks were sectioned at 4 μm thickness. Then mounted on positively charged slides, deparaffinized in xylene, and rehydrated through descending grades of ethanol. Antigen retrieval was performed by heating sections in citrate buffer (pH 6.0) using a water bath for 15–20 min, followed by cooling at room temperature. Hydrogen peroxide (3%) was used to inhibit endogenous peroxidase activity for ten minutes, and a protein blocking solution was used to reduce nonspecific binding. After that, sections were incubated with a primary rabbit polyclonal anti-Cas-3 antibody for a whole night at 4 °C^27^ and mouse monoclonal anti–TNF-α antibody^28^ at the manufacturer’s recommended dilution. Slides were treated with a biotinylated secondary antibody and then streptavidin–horseradish peroxidase (HRP) reagent following PBS washing. DAB (3,3′-diaminobenzidine) was used to visualize the immunoreaction, as the chromogen, producing a brown cytoplasmic staining, and the slides were counterstained with Mayer’s hematoxylin, dehydrated, and mounted. Cas-3 and TNF-α-positive cells were evaluated under a light microscope (Eclipse E800, Nikon) and photographed using an Olympus camera.

### Image analysis

For morphometric analysis, the mean area % of immunopositive expressions of Cas-3 and TNF-α activation were calculated in five non-overlapping randomly selected fields in five tissue paraffin sections among five rats in each group. The area % immunoexpression were analyzed with ImageJ software (ImageJ 1.54 g, National Institutes of Health, USA).

### Statistical analysis

SPSS for Windows (Version 18.0, Chicago, Illinois) statistics program for research was used to conduct the statistical analysis. Tukey’s multiple range test and post hoc analysis were employed in a one-way ANOVA to identify significant differences between experimental groups^29^. To present the findings, the means and standard errors of the means (SEMs) were computed. A *P value* of less than 0.05 was considered significant.

## Results

There were no deaths or adverse symptoms across the groups during the trial period.

### Changes in the lipid profile

Table 2 shows the lipid profile means and standard errors values for the various groups. The level of TG, TC, LDL-C were markedly increased and the level of HDL-C was markedly decreased in the rats that were received PFF (G3). While after 4 weeks of the investigation, co-treatment of the PFF group with omega-3 revealed significant reductions in TG, TC, and LDL-C levels. However, there were no appreciable variations in the HDL-C level between the PFF treated group (G3), and the group treated with both PFF and omega-3 (G4).

**Table 2:** Effect of omega-3 versus profenofos on the lipid profile in the various study groups after 4 weeks of treatment (mean±standard error), (*n* = 7).

	Control	Omega-3	Profenofos	Omega-3 + profenofos
TG (mg/dl)	34.212 ± 0.952^c^	35.322 ± 1.363^c^	89.568 ± 2.488^a^	61.849 ± 3.507^b^
TC (mg/dl)	34.806 ± 1.440^c^	32.982 ± 1.901^c^	72.163 ± 1.496^a^	54.863 ± 1.670^b^
HDL-C (mg/dl)	42.632 ± 1.186^a^	41.602 ± 1.125^a^	19.964 ± 0.746^b^	23.022 ± 0.527^b^
LDL-C (mg/dl)	40.552 ± 2.985^c^	36.580 ± 1.775^c^	78.797 ± 1.436^a^	59.594 ± 0.677^b^

Means with different superscripts in the same row are significantly different at *p* < 0.05.

### Changes in cholinesterase activity

Table 3 shows the cholinesterase activity means and standard errors values for the various groups. The rats that administered PFF had significantly lower levels of cholinesterase activity. While after 4 weeks of the investigation, co-treatment of the PFF group with omega-3 (G4) revealed significant increase in the cholinesterase activity.

**Table 3:** Effect of omega-3 versus profenofos on cholinesterase activity in the various study groups after 4 weeks of treatment (mean±standard error), (*n* = 7).

	Control	Omega-3	Profenofos	Omega-3 + profenofos
Cholinesterase activity (U/L)	3027.857 ± 17.808^a^	3021.321 ± 9.928^a^	2573.879 ± 36.539^b^	2939.071 ± 16.434^a^

Means with different superscripts in the same row are significantly different at *p* < 0.05.

### Changes in the brain oxidative markers

The oxidative indicators’ means and standard errors for each group are displayed in Table 4. Compared to the control and omega-3 groups, rats (G3) that received PFF exhibited considerably greater amounts of MDA in their brain tissues. After four weeks of the trial, rats treated with both omega-3 and PFF (G4) showed a considerable reduction in MDA levels. Similarly, compared to the control and omega-3 groups, the rats in G3 that received PFF had significantly reduced levels of GSH content and SOD activity. However, following four weeks of the trial, rats treated with both omega-3 and PFF (G4) showed a significant increase in GSH content and SOD activity.

**Table 4:** Effect of omega-3 versus profenofos on oxidative markers in the cerebral cortex homogenates of various study groups after 4 weeks of treatment (mean±standard error), (*n* = 7).

	Control	Omega-3	Profenofos	Omega-3 + profenofos
MDA nmol/mg protein	52.045 ± 2.139^c^	45.458 ± 4.981^c^	483.239 ± 18.589^a^	145.201 ± 10.713^b^
GSH µg/mg protein	0.399 ± 0.075^a^	0.449 ± 0.079^a^	0.051 ± 0.009^b^	0.188 ± 0.012^a^
SOD U/mg protein	180.801 ± 6.014^a^	192.226 ± 4.090^a^	28.541 ± 4.504^c^	104.547 ± 6.969^b^

Means with different superscripts in the same row are significantly different at *p* < 0.05.

### PI3K, AKT, NFκB, and STAT-3 mRNA gene expression in brain tissue

**Fig. 1:**
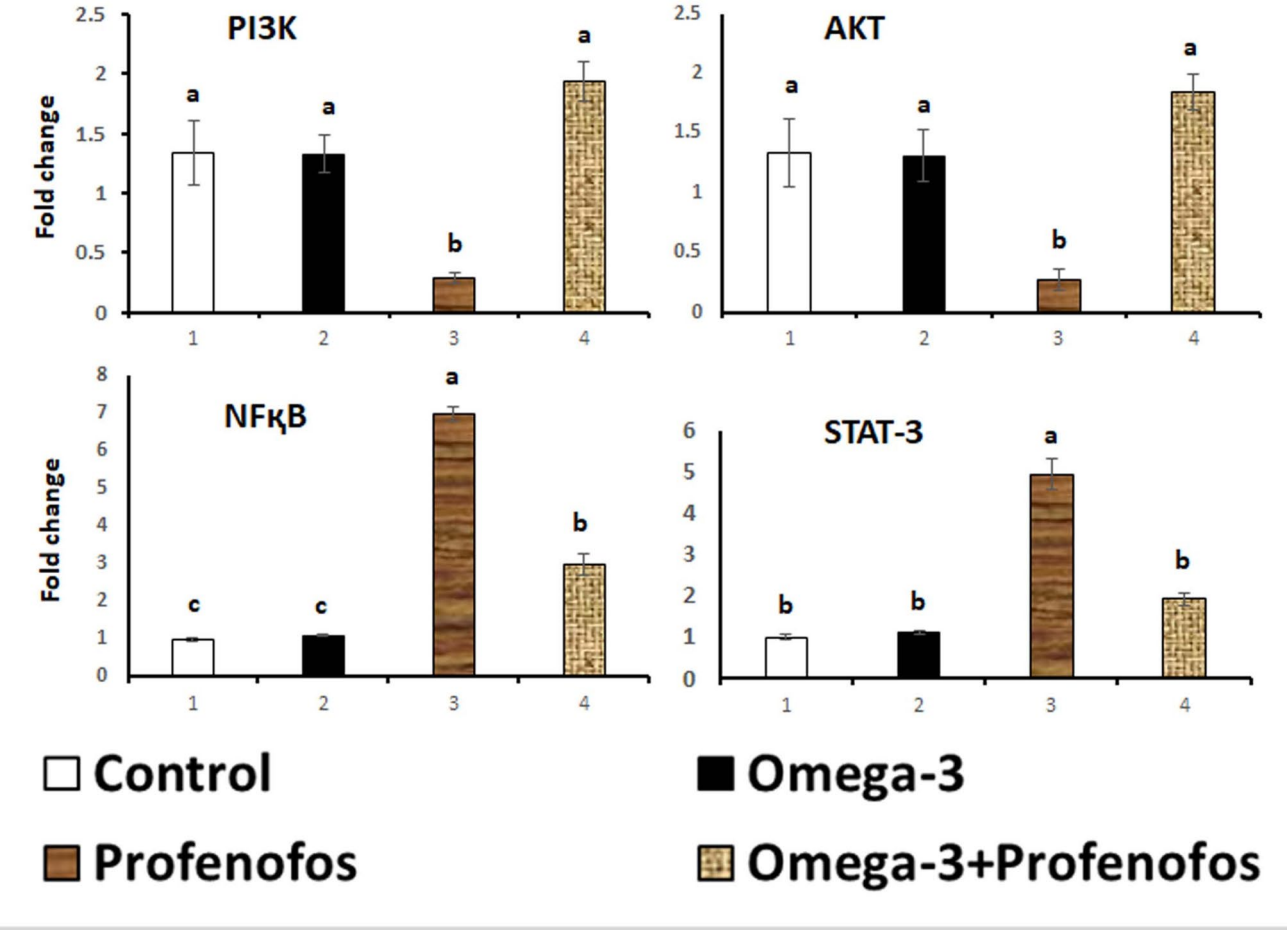
mRNA expression of brain PI3K, AKT, NFқB, and STAT-3. Total RNA was prepared from cerebral cortex of rats treated with omega-3, PFF, omega-3 against PFF, and control after 4 weeks of treatments. Real-time PCR was evaluated the expression levels. *p* < 0.05 compared with control values. Bars represent means± standard error (*n* = 7).

Figure 1 shows the expression of the PI3K, AKT, NFκB, and STAT-3 mRNAs in the cerebral cortex of the control, omega-3, PFF, and omega-3/PFF-treated groups after 4 weeks of treatment. NFκB and STAT-3 mRNA genes were significantly increased in the PFF treated rats, while PI3K and AKT levels were significantly downregulated. However, administering omega-3 to intoxicated rats resulted in a strong upregulation of PI3K and AKT mRNAs and a marked downregulation of NFκB and STAT-3 mRNAs.

### Histopathological assessment of brain tissue

**Fig. 2:**
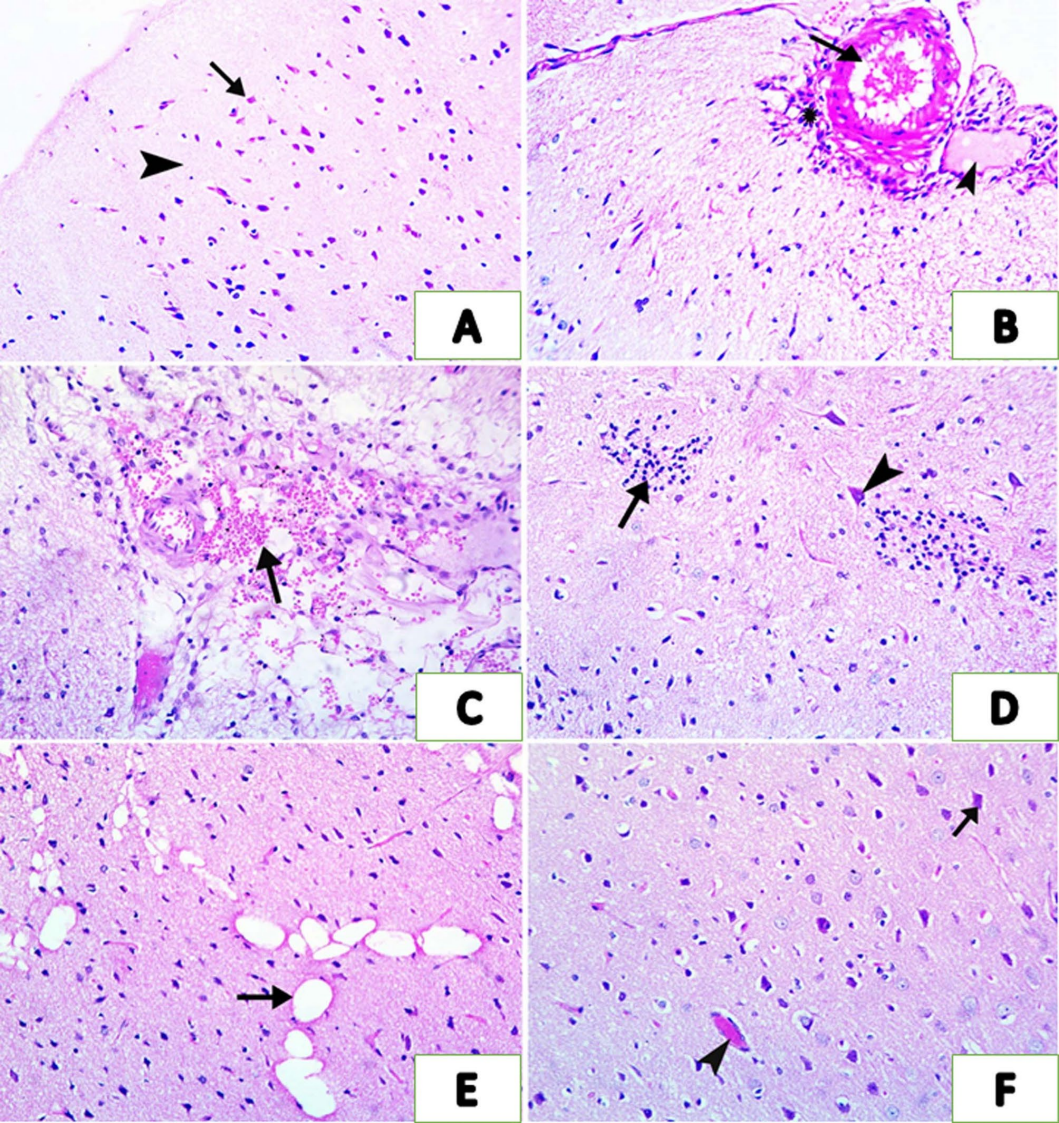
Representative photomicrographs of rat’s cerebral cortex sections in control, omega-3, profenofos (PFF), and PFF-omega-3 treated group. (**A**) The control group displaying normal cortical organization with neurons exhibiting vesicular nuclei, prominent nucleoli, and well-preserved cytoplasmic detail (arrow). The neuropil is compact and free of vacuolation (arrowhead), and blood vessels appear normal. (**B**–**E**) PFF-intoxicated group showing severe congestion of meningeal blood vessels (arrow) with perivascular edema (arrowhead) and leukocytic cellular infiltration (asterisk) (**B**), severe degeneration and necrosis of the cerebral cortex replacing by hemorrhage and edema (arrow) (**C**), focal glosis (arrow) and necrosis of some neurons (arrowhead) (**D**), spongiosis and vacuolation (arrow) (**E**). (**F**) PFF-intoxicated group treated with omega-3 revealing mild congestion of blood vessels (arrowhead), well-preserved neurons (arrow), and glial cells with minimal degeneration and necrosis. (H&E stain X200).

When cerebral cortex sections were examined, it became clear that intoxicated rats (G3) had cerebral blood vessels that were markedly congested (arrow) with prominent perivascular edema (arrow head) forming clear halos around capillaries and leukocytic cellular infiltration (asterisk) (Fig. 2B). Severe degeneration and necrosis of the cerebral cortex tissue replacing by hemorrhage and edema (arrow) (Fig. 2C). Neuronal degeneration was characterized by pyknosis, chromatolysis, and cytoplasmic eosinophilia (arrowhead). Reactive gliosis was evident, with hypertrophic glial cells and occasional neuronophagia, reflecting significant neuronal injury (arrow) (Fig. 2D). The neuropil demonstrated extensive vacuolation and spongiform changes, particularly in cortical and hippocampal regions (arrow) (Fig. 2E). Cotreatment of intoxicated rats with omega-3 resulted in mild congestion of blood vessels (arrowhead), well-preserved neurons (arrow), and glial cells with minimal degeneration and necrosis (Fig. 2F). The cerebral cortex tissues of rats in the control groups appeared to be normal; they exhibited cortical organization with neurons exhibiting vesicular nuclei, prominent nucleoli, and well-preserved cytoplasmic detail (arrow). The neuropil is compact and free of vacuolation (arrowhead), and blood vessels appear normal (Fig. 2A).

### Immunohistochemical assessment of brain tissue

**Fig. 3:**
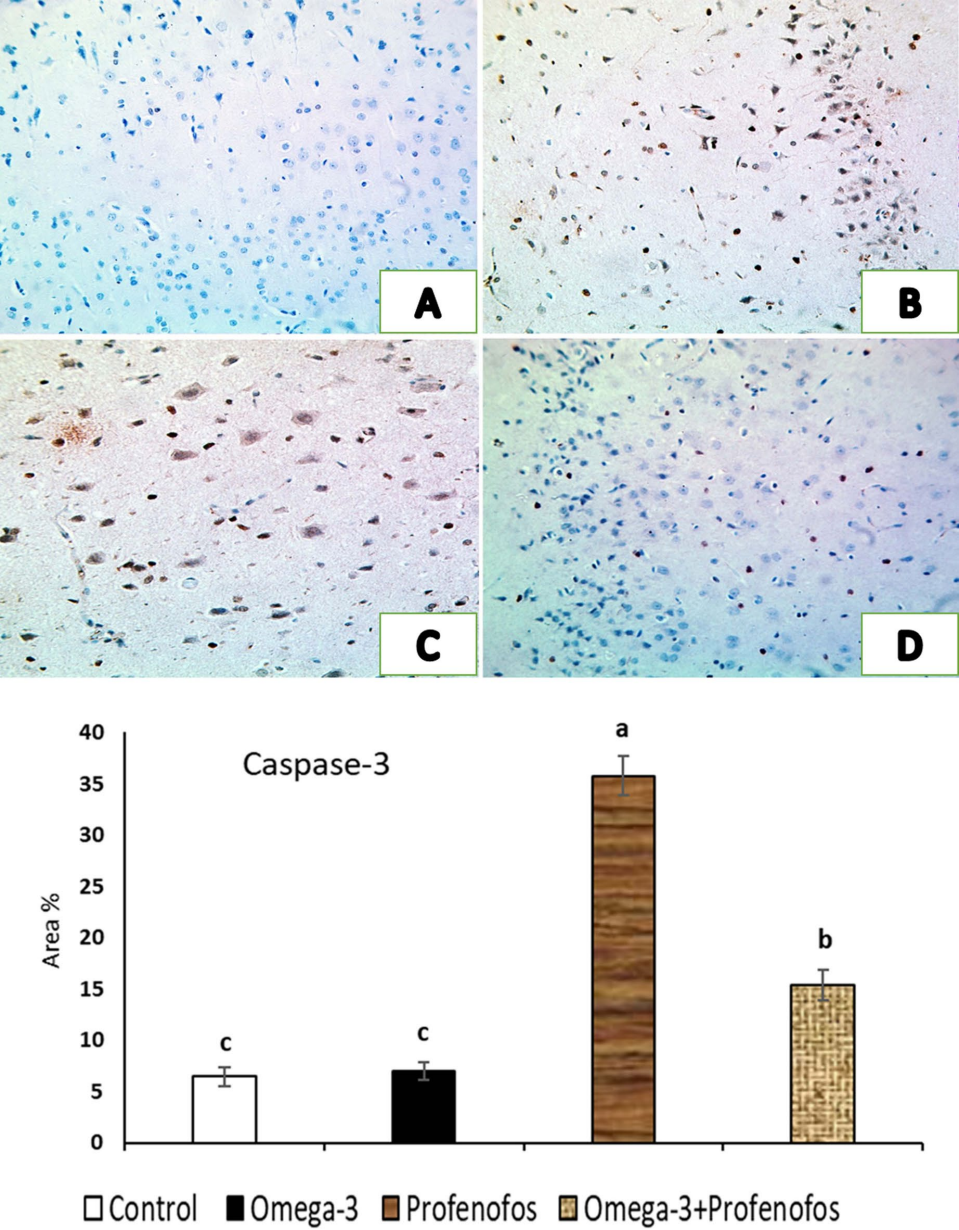
Representative photomicrographs showing the immunohistochemical expression of caspase-3 (Cas-3) in the cerebral cortex of rats x200, (**A**) Control group exhibiting a minimal immunoexpression of glial cells and neurons. (**B**,**C**) Profenofos (PFF) toxic group showing a very high immunoreactive response to Cas-3 with a marked increase in positive immunostained cells. (**D**) PFF-intoxicated group treated with omega-3 displaying marked decreases in the intensity of the brown color and the number of immunopositive cells. The quantitative analysis revealed that, area % of Cas-3 immunoreactivity in cerebral cortex of PFF-treated group was significantly higher than the control. Meanwhile, cotreatment of PFF group with omega-3 revealed significant decreases in area% of Cas-3 immunoreactivity.

**Fig. 4:**
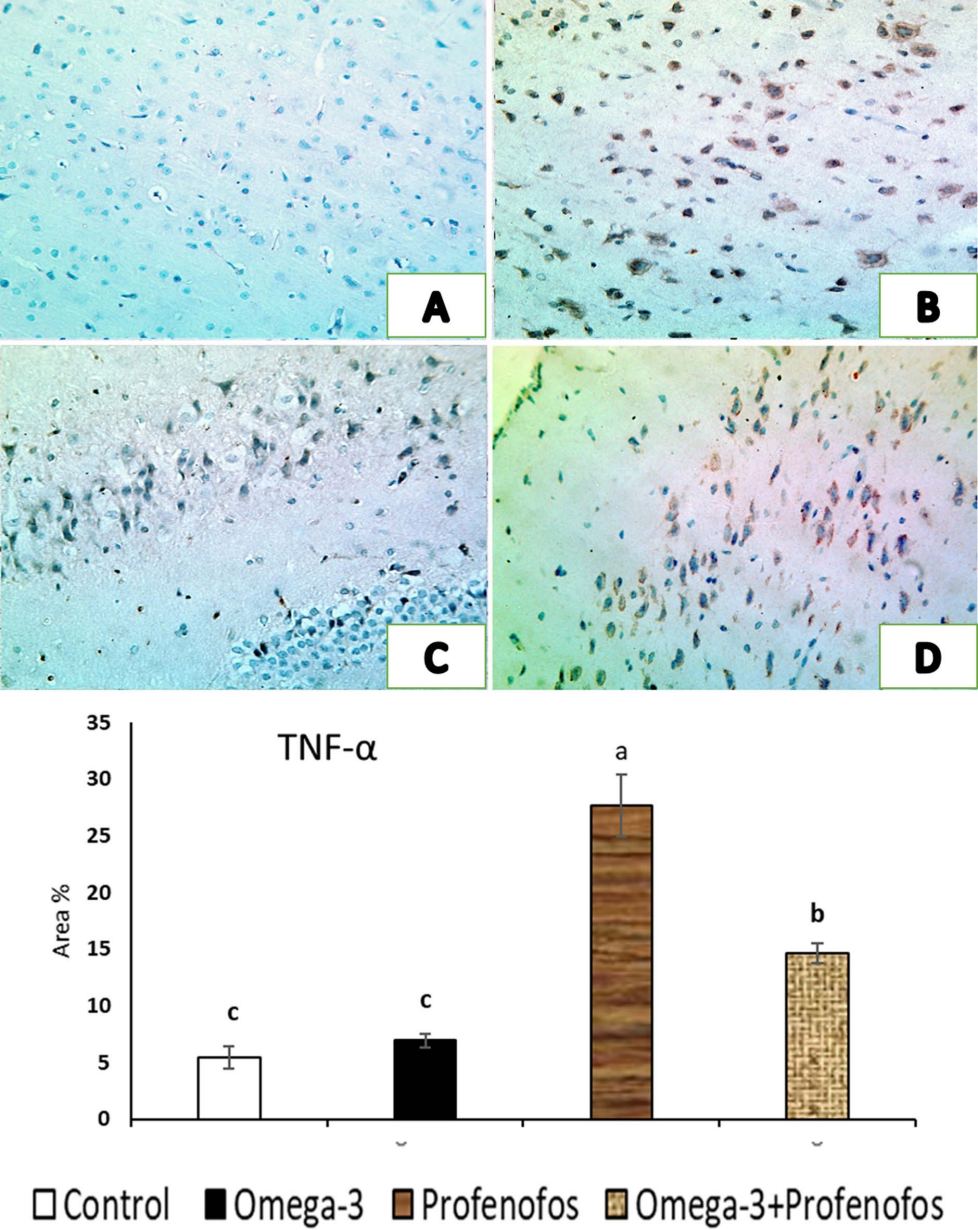
Representative photomicrographs showing the immunohistochemical expression of TNF-α in the cerebral cortex of rats x200, (**A**) Control group exhibiting absent or faint cytoplasmic staining and glial cells displaying minimal reactivity. (**B**,**C**) Profenofos (PFF) toxic group revealing a very high immunoreactive response to TNF-α with a marked increase in positive immunostained cells. (**D**) PFF-intoxicated group treated with omega-3 showing marked decreases in the intensity of the brown color and the number of immunopositive cells. The quantitative analysis revealed that, area % of TNF-α immunoreactivity in cerebral cortex of PFF-treated group was significantly higher than the control. Meanwhile, cotreatment of PFF group with omega-3 revealed significant decreases in area% of TNF-α immunoreactivity.

In cerebral cortex sections of the control, and omega-3 treated rats, Cas-3 and TNF-α immunostaining was negligible, with neurons showing absent or faint cytoplasmic staining and glial cells displaying minimal reactivity, consistent with normal physiological apoptosis levels (Figs. 3A and 4A). Meanwhile, the PFF-intoxicated group demonstrated a pronounced increase in Cas-3 and TNF-α immunoexpressing throughout the cerebral cortex, where numerous neurons exhibited intense brown cytoplasmic staining indicative of active apoptosis and inflammation, respectively. Strong positivity was also observed in the reactive glial cells, reflecting widespread neurodegeneration and apoptotic signaling (Fig. 3B, and C) and inflammation (Fig. 4B, and C) triggered by PFF toxicity. In contrast, the cotreatment of PFF intoxicated group with omega-3 had markedly lower Cas-3 (Fig. 3D) and TNF-α expression (Fig. 4D), with only a limited number of neurons displaying mild to moderate immunostaining, indicating a notable reduction in apoptotic glial cells and inflammation. Furthermore, the quantitative analysis (Fig. 3, and Fig. 4) revealed that, area % of Cas-3, and TNF-α immunoreactivity in cerebral cortex of PFF-treated group was significantly higher than the control. Meanwhile, cotreatment of PFF group with omega-3 revealed significant decreases in area% of Cas-3 and TNF-α immunoreactivity.

## Discussion

This investigation’s goal was to ascertain whether omega-3 fatty acids were sufficient to ameliorate the brain functioning of Wistar rats given PFF, which causes brain toxicity by targeting oxidative markers (MDA, GSH, and SOD), and gene expression (PI3K, AKT, NFқB, and STAT-3). Vegetables and fruits have been discovered to have traces of the moderately dangerous PFF^30^. The main route that humans are exposed to PFF is through the diet^31^. PFF’s leftovers residues into the surrounding air, surface water, and soil when it is sprayed to agricultural areas^32^. The rats in this trial showed no evidence of stress, and there were no clear clinical indications that PFF dose was detrimental.

TG, TC, and LDL-C values significantly increased in the G3, indicating hyperlipidemia. The adverse metabolic consequences could have been the cause of the higher levels of TG, TC, and LDL-C that resulted from oxidative stress of PFF. The ROS are produced by PFF in the liver and brain^33^. ROS cause lipid peroxidation, damaging cell membranes and disturbing lipid metabolism^34^. PFF induced its toxicity through impairs β-oxidation of fatty acids, lipoprotein synthesis and regulation, and cholesterol and triglyceride homeostasis^35^. High lipid levels trigger chronic inflammation that damages neurons, accelerates brain aging, and interfere with synapse formation and repair^36^. High LDL-C and triglycerides promotes beta-amyloid plaque buildup in brain arteries that can block blood flow, leading to brain ischemia. Chronic dyslipidemia damages tiny brain blood vessels leading to white matter damage^37^. A prior study found that chlorpyrifos induced a comparable increase in TG, TC, and LDL-C levels^38^.

The level of HDL-C was markedly decreased in the rats that were received PFF. HDL normally stabilizes tight junction proteins (claudin-5, and occludin) that reduces endothelial inflammation. Therefore, its deficiency increase blood brain barrier permeability and neuronal injury^39^. Low HDL-C leads to ApoA-I deficiency end to vasoconstriction, and increased platelet aggregation^32^. It also accelerate cholesterol accumulation in the brain leads to endothelial dysfunction^40^. Its deficiency leads to microglial over activation, oxidative stress that activate NFκB signaling. Low HDL-C reduced amyloid-β efflux that increased plaque deposition^41^.

Intoxicated rats treated with omega-3 showed a significant reduction in TG, TC, and LDL-C levels. The preventive action of omega-3 may have contributed to this result. According to a prior study, fenitrothion-induced hyperlipidemia could be lessened by fish oil^42^. By blocking sterol regulatory element-binding protein-1c (SREBP-1c), a crucial transcription factor for fatty acid synthesis, omega-3 reduces de novo lipogenesis in the liver. Additionally, it decreases the expression of acetyl-CoA carboxylase and fatty acid synthase leading to reduced hepatic triglyceride production, lowering very-low-density lipoprotein (VLDL) secretion. With lower TG availability and reduced synthesis, there is less VLDL produced by the liver; that directly reduces plasma TG levels^43^. Furthermore, Omega-3 activates the nuclear receptor peroxisome proliferator-activated receptor alpha (PPAR-α), which increases mitochondrial and peroxisomal β-oxidation of fatty acids and reduces the buildup of TG in the liver^44^. This promotes faster clearance of these lipoproteins from circulation^45^. A similar decrease in TG, TC, and LDL-C levels caused by omega-3 has been reported in a previous study^46^.

The present study revealed that cholinesterase activity significantly decreased in the PFF treated group that indicates neurotoxicity. This may have been due to phosphorylation of the serine hydroxyl group in the active site of the enzyme. This phosphorylation creates a stable enzyme-inhibitor complex that is resistant to hydrolysis. As a result, acetylcholine accumulates at synaptic junctions (nerve endings), especially at neuromuscular junctions, autonomic ganglia, and central nervous system synapses^47^. A prior study^3^ demonstrated a comparable decrease of cholinesterase activity mediated by PFF.

Intoxicated rats treated with omega-3 showed a notable improvement in cholinesterase activity. This result could have resulted from alterations to protein expression involved in cholinergic signaling. This includes potentially enhancing the activity of acetylcholinesterase^48^. Omega-3 fatty acids, particularly DHA, are incorporated into the phospholipid bilayers of neurons and other cells. This incorporation enhances membrane fluidity, which is important for the functionality of neurotransmitter receptors and to the activity of cholinesterase^49^. In a prior study, wistar rats and their pups showed a comparable recovery in cholinesterase activity due to omega-3^50^.

According to the current investigation, rats treated with PFF showed considerably higher MDA values and significantly lower GSH and SOD values, which indicated oxidative stress. PFF increases the generation of ROS, which attack membrane lipids leading to lipid peroxidation. This damages cellular membranes, affecting cell permeability, and membrane-bound enzymes^33^. PFF interferes with mitochondrial complexes, leading to impaired electron transport chain (ETC), leakage of electrons, and enhanced ROS formation. Mitochondrial damage further promotes apoptosis and necrosis^34^. PFF reduces the activity and levels of key antioxidant enzymes GSH, and SOD leading to imbalance between ROS and antioxidant defense. In a prior study, Nile tilapia (Oreochromis niloticus) treated to PFF showed comparable outcomes^51^.

Conversely, when intoxicated rats given omega-3, the levels of MDA decreased while the levels of GSH and SOD significantly increased. Omega-3 can increase the synthesis and recycling of GSH. This is crucial in detoxifying peroxides and maintaining redox balance in neurons^52^. The brain is known to benefit from the antioxidant properties of omega-3, especially DHA and EPA^11^. DHA and EPA also generate resolvins and protectins, which actively resolve ROS. DHA improves membrane fluidity, cell signaling, and resistance to oxidative stress. This helps stabilize neuronal membranes and protects them from oxidative damage^53^. DHA-derived metabolites (e.g., neuroprotectin D1) can directly reduce oxidative damage in neurons. It also improves mitochondrial function, stabilizes mitochondrial membranes, and reduces mitochondria-derived ROS^54^. These results are consistent with those of Hanie, et al.^55^, who found that omega-3 has antioxidant properties in male rats with dorsal hippocampal lesions.

In the present study, there was significant downregulation of brain PI3K, and AKT mRNA gene expression and significant upregulation of brain NFқB, and STAT-3 mRNA gene expression when rats treated with PFF. These findings agree with MATE et al.^10^. Cell growth, metabolism, and survival depend on the PI3K/AKT genes^8^. PFF inhibits PI3K and AKT phosphorylation. This making cells more susceptible to oxidative damage and apoptosis^56^. Inhibited AKT signaling enhancing mitochondrial dysfunction, and reduce cellular repair^56^. PFF can aberrantly activate NFκB, and STAT-3 possibly through ROS liberation. This leads to nuclear translocation of NFκB, and STAT-3 and transcription of pro-inflammatory and pro-apoptotic genes in the brain^9^. STAT-3 regulates cell growth, differentiation, inflammation, and survival. Aberrant activation of STAT-3 leads to disruption of redox homeostasis, and loss of protective cellular response^10^.

On the other side, brain PI3K, and AKT mRNA gene expression shown to be upregulation and brain NFқB, and STAT-3 mRNA gene expression shown to be downregulation in rats given omega-3 and PFF. These findings corroborate those of Darwish et al.^57^. The anti-inflammatory, antioxidant, and cell-protective activity of omega-3 particularly DHA and EPA are widely recognized^58^. Omega-3 reduces transcription of pro-inflammatory genes, and suppresses ROS-induced NFκB activation^59^. Omega-3 Modulates STAT-3 activation as it inhibits over activation of STAT-3 to reduce cytokine storms^57^.

These results validated the improvement in the quantitative analysis of area % of TNF-α and Cas-3 immunoreactivity in cerebral cortex of PFF-treated rats that was significantly higher than the control. These findings indicates active apoptosis and inflammation. Low HDL-C and oxidative stress activates TNF-α. TNF-α activates NFκB and STAT-3 genes to promote inflammation and survival. Chronic activation overwhelms protective mechanisms, leading to Cas-3-mediated apoptosis^60^. However, cotreatment of PFF group with omega-3 revealed significant decreases in area% of Cas-3 and TNF-α immunoreactivity, suggesting a significant decrease in inflammation and apoptotic glial cells. These results aligned with those published by Sajad et al.^61^.

These results demonstrated that the histological pictures of the cerebral cortex of rats treated with omega-3 were improved. PFF-intoxicated rats showed cerebral blood vessels congestion with prominent perivascular edema, leukocytic cellular infiltration, severe degeneration and necrosis of the cerebral cortex. These results were consistent with earlier studies^62^. However, administering omega-3 to intoxicated rats produced mild congestion of brain blood vessels, well-preserved neurons, and glial cells with minimal degeneration and necrosis. These results were consistent with Bi et al.^63^.

## Conclusion

The current study emphasized omega-3 as a possible neuroprotective medication against PFF-induced neurotoxicity. Thus, by directly scavenging ROS and blocking the enzymes that generate free radicals, omega-3 can lessen oxidative stress, enhancing the activity of antioxidant enzymes, inducing the transcription of PI3K/AKT genes, and inhibiting apoptotic and pro-inflammatory factors (Cas-3 and TNF-α).

### Limitation of the study

Further research is required to evaluate behavioral tests, and to assessment the protein levels.

## Data Availability

The corresponding author will supply the data supporting the study upon request.
